# Tolerability of endometriosis medical treatment: a comparison between combined hormonal contraceptives and progestins

**DOI:** 10.1186/s12905-023-02647-y

**Published:** 2023-09-23

**Authors:** Denise Joffily Pereira da Costa Pinheiro, Ana Maria Gomes Pereira, Marcelo Antonini, Isabella Maria Albuquerque Salgado, Alexandre Torchio Dias, Reginaldo Guedes Coelho Lopes

**Affiliations:** 1 Hospital Do Servidor Público Estadual - Francisco Morato de Oliveira (HSPE-FMO), Rua Pedro de Toledo 1800, São Paulo, SP 04029000 Brazil; 2 CEDEP – Instituto de Assistência Médica ao Servidor Público Estadual (IAMSPE), São Paulo, Brazil

**Keywords:** Endometriosis, Pharmacological treatment, Side effects, Tolerability

## Abstract

Endometriosis is a chronic inflammatory disease that occurs in women of reproductive age. Much of the treatment involves hormone therapy that suppresses the proliferation of endometriosis lesions.

**Objective** To compare discontinuation rates of pharmacological treatment with estrogen-progestins and progestins medications. The secondary objective is to evaluate the main side effects of these drugs in patients with endometriosis.

**Methods** This retrospective study analyzed data from 330 patients who attended the Hospital of the State Public Servant of São Paulo from August 1999 to September 2020 and received pharmacological treatment for endometriosis. The data were obtained by review of the files of medical appointments with specialized staff.

**Results** The median treatment time was 18 months, ranging from 1 to 168 months, and 177 patients interrupted the proposed treatment. The combined contraceptives with estrogens and progestins were significantly linked to treatment interruption, with a relative risk of 1,99 (*p* = 0,005). The most important side effects that resulted in treatment interruption were pain persistence (*p* = 0,043), weight gain (*p* = 0,017) and spotting (*p* < 0,001).

## Introduction

Endometriosis is a chronic inflammatory estrogen-dependent gynecological disease characterized by the development and growth of functional endometrium-like tissue outside the uterine cavity [1, 2]. It predominantly affects the ovaries but can also affect other organs such as the fallopian tubes, pelvic ligaments, appendix, bladder, and intestines [3–5].

The most common symptoms are dysmenorrhea, pelvic pain outside the menstrual period, dyspareunia, infertility, urinary and evacuation symptoms. However, its clinical presentation can be non-specific and with symptoms disproportionate to the extent of the disease, making diagnosis difficult [6–10].

Endometriosis significantly impacts women's quality of life, compromising their social and emotional relationships, work, and study performance. It is an important public health issue, affecting 6 to 10% of women of reproductive age, with a peak incidence between the ages of 25 and 35 years [4, 5].

The treatment of endometriosis includes surgery, medication therapy, and assisted reproductive techniques. As a chronic disease, patients should be monitored for many years and receive individualized treatment according to their clinical status and reproductive desire at each stage of life. The goal is to remove endometriotic foci surgically or suppress them with clinical treatment. However, the best approach has not been defined yet [11–14].

The medical treatment aims to induce a hypoestrogenic state of chronic anovulation, creating an inadequate environment for the growth and maintenance of endometriosis implants [7, 13, 15, 16]. The medical treatment is not curative, as it cannot eliminate the endometriotic foci, only making them temporarily inactive during medication use [17].

Among the therapeutic options, we have combined hormonal contraceptives containing estrogens and progestins (EP), isolated progestins (P), antiprogestins, GnRH agonists, GnRH antagonists, aromatase inhibitors, and medications that do not act as hormonal suppressants, such as analgesics and non-steroidal anti-inflammatory drugs [9].

Considering the chronic use of these medications, it is important to evaluate not only their efficacy but also their tolerability, side effects, cost, and each patient's preferences [7, 13, 17, 18]. The tolerability of treatment consists of the patient's ability to tolerate the side effects and maintain the use of the medication. It can be evaluated through the rates of treatment interruption or follow-up losses in clinical studies [17].

It is recommended to start with low-cost drugs, such as combined oral contraceptives and some progestins, and then move on to high-cost drugs, such as GnRH agonists, in cases of low adherence, tolerability, or ineffectiveness [7].

Although widely prescribed, combined hormonal contraceptives have no scientific basis to prove the superiority of this group of medications compared to other classes, and does not appear to be any advantage of any specific drug within this group [7]. Continuous administration of combined contraceptives has been more favorable in controlling pain than cyclical administration. It is possible to perform a planned interruption only to control spotting, which is bleeding that occurs outside of the menstrual period [19]. Regarding ethinylestradiol dosage, low-dose options with 20 mcg are safer, with a lower risk of thromboembolic events [17].

According to some authors, progestins have fewer side effects than combined contraceptives and can be prescribed in various routes of administration, oral, injectable, implants, and intrauterine devices [9, 20–25]. Desogestrel and dienogest are 19-nortestosterone-derived progestins widely studied for the treatment of endometriosis and have been shown effective in controlling symptoms [20, 22].

Symptoms can be controlled by various drugs, many of them with great pain control results, the limiting factors are the side effects and tolerability related to these medications. Adequate monitoring and control of unwanted effects are essential for achieving therapeutic success. Thus, studies that compare drug options, considering not only the efficacy but also the quality of life of patients, are necessary to guide conduct.

## Objectives

This study aims to compare the discontinuation rates of medical treatments for endometriosis with combined hormonal contraceptives and isolated progestins. The secondary objective is to evaluate the main adverse effects related to the discontinuation of these medications.

## Methods

A retrospective study that evaluated the rate of medication interruption by patients attended in the endometriosis sector of the State Public Servant Hospital in São Paulo.

The data was collected through forms filed in the specialized outpatient clinic. Patients attended from August 1999 to September 2020 were evaluated.

To be included in the study, a histological confirmation of endometriosis and a medical treatment prescription was necessary. Thus, it is important to highlight that all patients included in the study underwent surgical treatment prior to clinical intervention. Patients with incomplete data for the study, those who were already in clinical or surgical postmenopause at the first consultation, hysterectomized patients and finally, those patients who did not have a minimum follow-up time of 6 months in the presence of medical treatment were excluded.

Epidemiological data were collected to trace the profile of attended patients.

The time between the onset of symptoms and the surgery date was evaluated. The symptoms questioned were dysmenorrhea, dyspareunia, cyclic pain, pain while evacuating, pericicatrical pain, infertility, urinary and intestinal symptoms.

The surgical findings were raised, researching where endometriotic lesions were found. The surgical procedures performed and the staging of endometriosis were also researched. The classification of endometriosis from the American Society for Reproductive Medicine (ASRM) was used as a reference [8].

The prescribed medications for clinical treatment were chosen based on reliable guidelines such as ESHRE’s, on the opinion of the attending physician and on the patient’s preferences [6]. These treatments were verified for the type of hormone and dose. During the entire follow-up, patients were questioned about the symptoms and side effects presented during treatment. The use time of each medication was recorded, and once the patient opted for discontinuation, the reason for discontinuation was also recorded. A new medication could be prescribed, containing the same hormone with a different dosage or a medication from a different class.

An informed consent form was applied before data collection. This study was approved by Research Ethics Committee of State Public Servant Hospital and is registered in Plataforma Brasil under CAAE number 36271213.8.0000.5463.

Statistical analyzes were performed for two distinct groups, isolated progestins (P) and combined contraceptives (EP). Frequencies were calculated using the information available for each data point.

The data obtained were grouped in an Excel spreadsheet for Windows® and were analyzed using the statistical programs Epi Info7® an Open-Epi, online version [26]. Continuous variables were tested for their distribution and are presented in means and standard deviation or medians and quartiles, depending on the normality of this distribution. Categorical variables are presented in percentages, according to the data available for analysis.

The variables relating to adverse events and complaints reported during clinical treatment were correlated with the outcome of treatment interruption, and multiple analysis of logistic regression was conducted using the STATA 12.0® program, grouping the adverse events that presented potential statistical significance in the univariate analysis (value of *p* < 0.25 was used to select variables for multiple final analysis), except those that did not have enough outcome events to be included in the adjusted modeling.

A *p*-value < 0,05 was considered statistically significant.

## Results

**Fig. 1:**
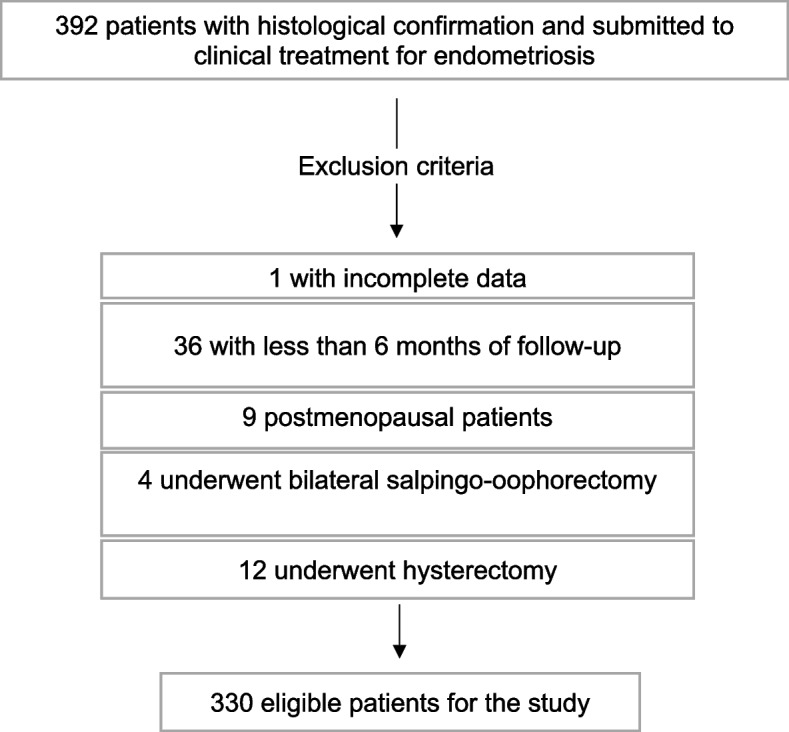
Flowchart of patients attended by the Endometriosis sector of the State Public Servant Hospital in São Paulo

As reported in Fig. 1, we enrolled 392 patients in the study, and after applying exclusion criteria, 330 patients remained for analysis.

The average age of patients at the time of the first consultation was 37.57 years (± 6.27), ranging from 17 to 53 years. The average age of symptom onset was 31.07 years (± 8.4), ranging from 8 to 51 years.

The average age of menarche was 12.46 years (± 1.73), ranging from 8 to 17 years. Out of the total, 138 patients (42.72%) had no children at the time of the first consultation and 40 patients (12.39%) had had one or more abortions.

Hormonal contraceptive methods were used by 145 patients (46.17%), 36 (11.46%) used permanent methods, and 96 (30.57%) used only condoms or no method. Three patients were using GnRH analogs at the time of the first consultation. Table 1 describes the most common conditions; arterial hypertension was the most frequent.

**Table 1:** Personal medical history reported by patients at the first visit

Comorbidities	Frequency	Percentage
**Cancer**	7	2.12%
**Cardiopathies**	10	3.03%
**Diabetes Mellitus**	16	4.85%
**Arterial Hypertension**	43	13.03%
**Thyroidopathies**	17	5.15%
**Gynecological Diseases**	20	6.06%
**Psychiatric Diseases**	0	0%

**Fig. 2:**
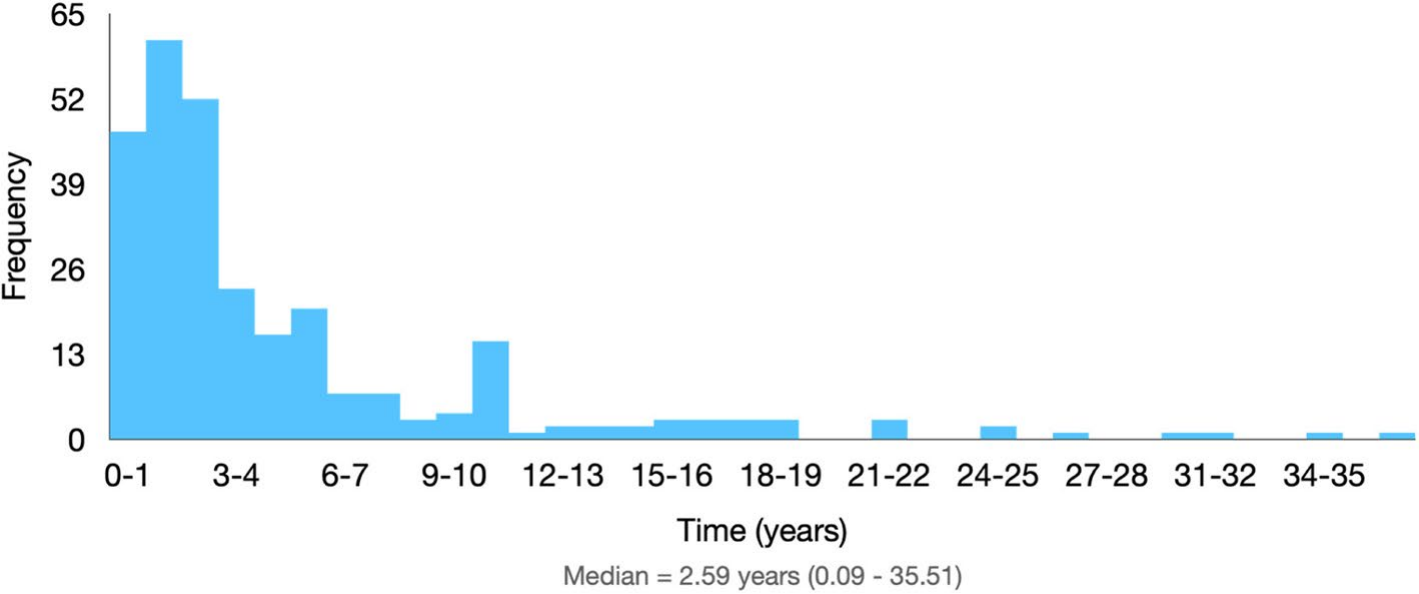
Time elapsed between onset of symptoms and surgical diagnosis

Of the total, 18 patients (5.45%) were asymptomatic at the time of the first consultation and 3 (0.91%) received the diagnosis in surgeries indicated by other hypotheses, making endometriosis a surgical finding. Twenty-five patients (7.57%) did not have data for calculating the time of symptoms. Thus, 284 patients remained, with whom it was possible to calculate the time between the onset of symptoms and the diagnosis. The median time between symptoms and surgical diagnosis of endometriosis was 31.1 (14.13—63.53) months, ranging from 1.03 to 426.13 months, data illustrated in Fig. 2.

Table 2 illustrates the symptoms reported by the patients before starting prescribed treatment. In Table 3, it is possible to evaluate the main sites of endometriotic lesions described in surgical reports.

**Table 2:** Symptoms reported by patients at the first appointment

Symptoms	Frequency	Total Available	Percentage
**Dysmenorrhea**	266	329	80.85%
**Dyspareunia**	144	327	44.04%
**Acyclic Pain**	144	329	43.77%
**Painful Defecation**	13	329	3.95%
**Pericicatricial Pain**	14	329	4.26%
**Infertility**	61	326	18.71%
**Hematuria/Urinary Symptoms**	8	328	2.44%
**Constipation**	102	320	31.88%
**Intestinal Bleeding**	2	328	0.61%
**Tenesmus**	5	329	1.52%

Percentages are calculated based on available data

**Table 3:** Location of endometriotic lesions

Location	Frequency	Total Available	Percentage
**Ovaries**	214	326	65.64%
**Rectovaginal Septum**	9	326	2.76%
**Rectum and Sigmoid Colon**	19	326	5.83%
**Bladder**	9	326	2.76%
**Appendix**	4	57	7.02%
**Abdominal Wall**	22	57	38.60%
**Fallopian Tube**	22	57	38.60%
**Retrocervical Region and Uterosacral Ligament**	22	228	9.63%

Percentages are calculated based on available data

According to the available surgical descriptions, 74 (48.05%) capsule resections, 40 (25.81%) oophorectomies, 37 (23.87%) cyst drains, and 13 (8.44%) cauterizations were performed.

We obtained the description of the pelvic endometriosis stage according to the American Society for Reproductive Medicine (ASRM) classification for 248 patients, distributed as follows: 21 (8.47%) cases of minimal endometriosis, 26 (10.48%) cases of mild endometriosis, 90 (36.29%) cases of moderate endometriosis, 103 (41.53%) cases of severe endometriosis, and 8 patients with a diagnosis of abdominal wall endometriosis (3.23%). Thus, 77.82% of the cases were in stages III or IV and 18.95% of the cases were in stages I or II.

All patients included received drug treatment according to Table 4 below. These methods were studied according to composition, isolated progestins (P) or combinations of estrogens and progestins (EP) to facilitate data interpretation.

**Table 4:** Medications prescribed at the start of the follow-up

Medication	Frequency	Percentage
**Transdermal EP**	1	0.30%
**Oral EP**	137	41.52%
**Vaginal EP**	4	1.21%
**GnRH Analogs**	4	1.21%
**IUD P**	15	4.55%
**Injectable P**	73	22.12%
**Subcutaneous P**	1	0.30%
**Oral P**	95	28.79%
**Total**	330	100%

*EP* combined hormonal contraceptives, *P* progestins

Therefore, 4 patients (1.21%) received GnRH analogs as the first option of medical treatment, 142 patients (43.03%) received combined methods prescriptions, and 184 patients (55.76%) received a prescription for isolated progestins. The preferred prescription form was continuous.

**Fig. 3:**
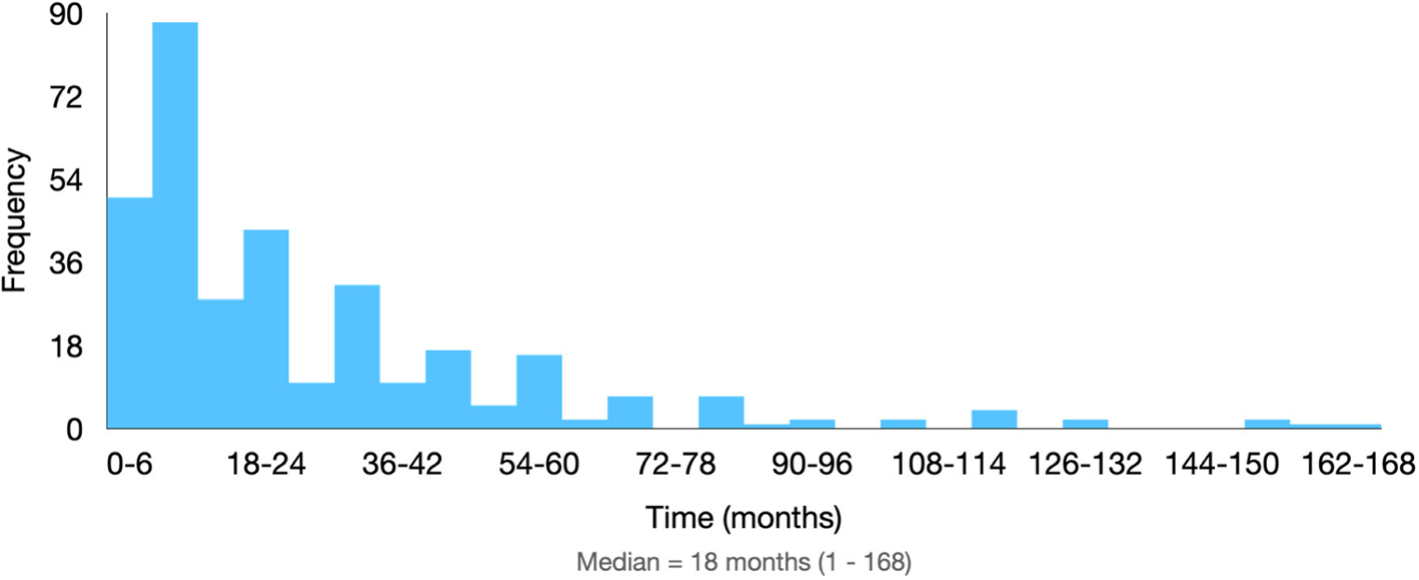
Time of use of the medication proposed as the initial treatment

Among the combined contraceptives, the most frequently prescribed dose was 30 µg of ethinylestradiol, prescribed for 95 patients (28.78%). Among the isolated progestins, the most frequent was desogestrel, prescribed for 87 (26.36%) patients. As shown in Fig. 3, the median treatment time was 18 months, ranging from 1 to 168 months. Of the total, 177 patients (53.63%) discontinued the proposed treatment.

During de follow-up after starting treatment, the patients reported several complaints, as shown in Table 5.

**Table 5:** Symptoms reported in follow-up consultations after starting the first proposed treatment

Symptoms	Frequency	Percentage
**Headache**	11	3.33%
**Persistence of Pain**	110	33.33%
**Spotting**	164	47.9%
**Intense Bleeding**	14	4.24%
**Breast Pain**	3	0.91%
**Nausea**	7	2.12%
**Weight Gain**	24	7.27%

**Fig. 4:**
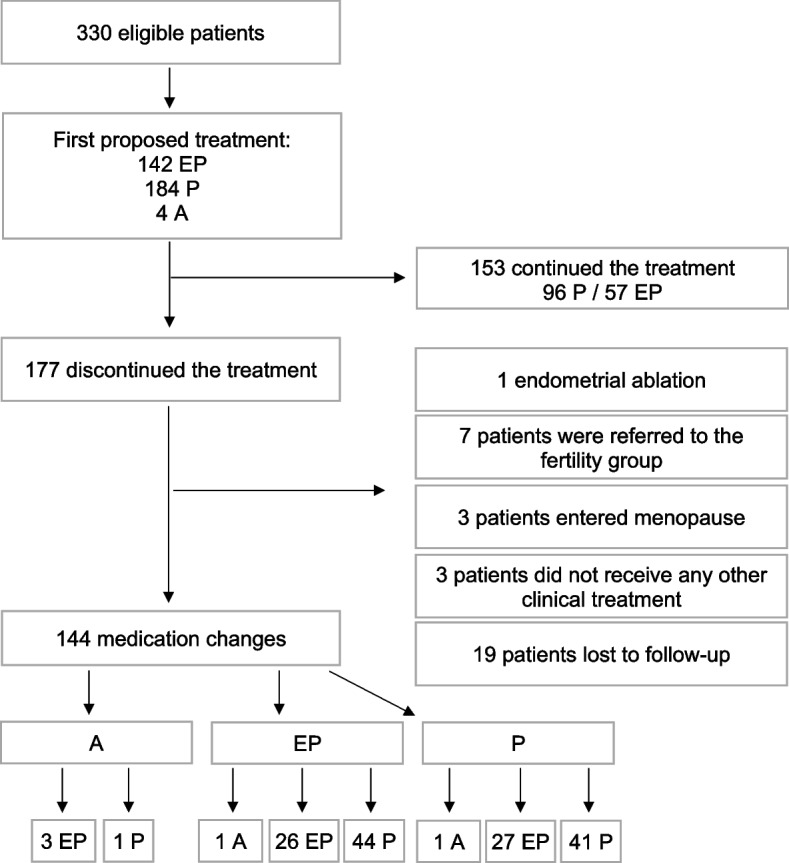
Flowchart of medication treatment and evolution. EP Estrogen-progestins, P progestins, A GnRH analogs

Figure 4 illustrates the evolution of the patients monitored during the study.

Out of the total, 153 patients continued with the initially prescribed medication, while 177 discontinued treatment. Among those who discontinued, 11 did so for reasons unrelated to treatment dissatisfaction, 3 chose not to receive medical treatment, and 19 were lost to follow-up after the initial 6 months, which were used as inclusion criteria for the study. The remaining 144 patients were prescribed a new medication.

Considering the patients who maintained the medication and those who required a treatment change, we obtained a discontinuation rate of 55.4% among EP users and 41.8% among P users.

Analyzing only the patients who discontinued the use of medication, based on the reported side effects, we obtained headache in six patients (9.84%), breakthrough bleeding in 47 (77.08%), weight gain in eight (13.12%), persistence of pain in 23 (37.72%), nausea in six (9.84%), mastalgia in two (3.28%) and acne in one patient (1.64%).

Given the complaints reported and the interruption of treatments, medication changes were proposed. Of these, 67 changes (46.52%) were made to medications in the same category and 77 changes (53.47%) to medications in a different category, as shown in Table 6.

**Table 6:** Medication swaps after the first proposed treatment

Medication Changes	Frequency	Percentage
**From EP to EP**	26	18.06%
**From EP to A**	1	0.69%
**From EP to P**	44	30.57%
**From A to EP**	3	2.08%
**From A to P**	1	0.69%
**From P to EP**	27	18.75%
**From P to A**	1	0.69%
**From P to P**	41	28.47%
**Total**	144	100%

Source: the author (2022)

*EP* combined hormonal contraceptives, *P* progestins, *A* GnRH analog

Six medication changes involved GnRH agonists. Of the four patients who started the follow-up with GnRH agonists, three switched to combined hormonal contraceptives and one to progestins. Two patients changed to GnRH agonists, one used EP and the other P, previously.

Of the 153 patients who did not interrupt the initial proposed treatment, 96 received P and 57 received EP. The average follow-up time for these patients was 38.91 months.

Among the users of EP, the events that showed an association with the interruption of treatment were persistence of pain, with a relative risk of 1.65 (*p* = 0.031) and breakthrough bleeding, with a relative risk of 2.76 (*p* < 0.001). All of this group who reported weight gain interrupted the treatment, but there was no statistical significance due to the low frequency of this complaint (*p* = 0.1345). It was observed that the highest risk of interruption of EP occurs up to 9 months of treatment, with a relative risk of interruption of 2.32 (*p* = 0.026). Treatment time above 10 months did not correlate with the risk of interruption.

Among the users of P, the events that had a greater impact on the risk of interrupting treatment were breakthrough bleeding and heavy bleeding, with a relative risk of 1.35 (*p* = 0.032). The time required for adaptation to treatment, and consequently not showing a correlation with medication interruption, was longer for P users. Up to 84 months of treatment, we have a relative risk of interruption of 1.74 (*p* = 0.04), becoming non-significant thereafter.

When the two groups were compared, the patients who received P as the initial treatment had a significantly higher age range (*p* < 0.001) and had some reported personal medical history (*p* < 0.001) compared to those who received EP.

The multiple logistic regression analysis that correlated adverse events, type of medication, and treatment interruption showed that the complaint of breakthrough bleeding, weight gain, persistence of pelvic pain, and treatment with EP had a direct, significant, and independent association with clinical treatment interruption, adjusted for the complaint of headache, as shown in Table 7.

**Table 7:** Multiple analysis of side effects, type of medication used, and treatment interruption

Treatment Type and Side Effect	"*p*" Value of Univariate Analysis	Univariate Analysis OR (CI)	"*p*" Value of Adjusted Analysis	Adjusted Analysis OR (CI)
**Spotting**	< 0.001	2.267 (1.457–3.528)	< 0.001	2.672 (1.672–4.269)
**EP**	0.016	1.725 (1.106–2.692)	0.005	1.995 (1.237–3.219)
**Persistence of Pain**	0.043	1.619 (1.015–2.582)	0.043	1.670 (1.017–2.743)
**Weight Gain**	0.066	2.330 (0.946–5.740)	0.017	3.227 (1.237–8.418)
**Headache**	0.080	3.994 (0.849–18.778)	0.076	4.440 (0.855–23.060)

*OR* Odds Ratio, *CI* confidence interval, *EP* combined contraceptives containing estrogens and progestins

Considering only the patients who received EP, the multiple analysis showed that the adverse events of spotting and persistence of pain and the staging of minimal/mild endometriosis had a significant, direct and independent correlation with the interruption of treatment with EP. Infertility had an inverse correlation with the interruption of treatment. These data are shown in Table 8. The variables were adjusted for a treatment duration of fewer than 9 months, education level, and complaint of headache.

**Table 8:** Multivariate analysis of the correlation between side effects, education, staging, and follow-up time with the risk of interrupting EP treatment

Side Effect	"*p*" Value of Univariate Analysis	Univariate Analysis OR (CI)	"*p*" Value of Adjusted Analysis	Adjusted Analysis OR (CI)
**Spotting**	< 0.001	4.267 (2.028–8.978)	< 0.001	8.432 (2.632–27.014)
**Persistence of Pain**	0.117	1.769 (0.898–3.610)	0.006	6.388 (1.721–23.714)
**Infertility**	0.136	0.533 (0.234–1.218)	0.010	0.153 (0.037–0.632)
**Duration of Treatment Less than 9 Months**	0.041	3.291 (1.048–10.335)	0.067	4.550 (0.8990–23.009)
**Staging Minimal/Mild**	0.155	2.106 (0.755–5.874)	0.026	5.578 (1.233–25.233)
**University Degree**	0.236	0.636 (0.331–1.344)	0.429	0.619 (0.188–2.032)
**Headache**	0.299	2.348 (0.469–11.742)	0.435	2.741 (0.217–34.491)

*EP* combined contraceptives containing estrogens and progestins, *OR* Odds Ratio, *CI* confidence interval

As shown in Table 9, the adverse event that had a significant and independent correlation with treatment interruption with P was the presence of spotting. There was also a direct correlation with duration of treatment less than 9 months and intraoperative endometriosis staged as minimal or mild.

**Table 9:** Multivariate analysis of the correlation between side effects, stage, and parity with the risk of treatment interruption with P

Side Effect	"*p*" Value of Univariate Analysis	Univariate Analysis OR (CI)	"*p*" Value of Adjusted Analysis	Adjusted Analysis OR (CI)
**Duration of Treatment Less than 9 Months**	< 0.001	5.625 (2.410–13.130)	< 0.001	9.398 (3.006–29.378)
**Spotting**	0.057	1.775 (0.984–3.203)	0.024	2.638 (1.134–6.136)
**Weight Gain**	0.073	2.405 (0.923–6.271)	0.211	2.110 (0.655–6.796)
**Intense Bleeding**	0.086	4.062 (0.821–20.104)	0.308	3.443 (0.319–37.174)
**Staging Minimal/Mild**	0.005	5.206 (1.654–16.382)	0.001	8.078 (2.291–28.482)
**Nulliparity**	0.171	1.538 (0.830–2.852)	0.239	1.667 (0.711–3.907)

*P* progestins, *OR* Odds Ratio, *IC* confidence interval

Considering that the systemic exposure to levonorgestrel among LNG-IUS users is minimal, a multivariate analysis of the P group was conducted, excluding those patients who were prescribed LNG-IUS as the initial treatment. This analysis did not find significant differences compared to the results presented in Table 9.

## Discussion

The medication therapy for endometriosis consists of long-term treatment, like therapies for other chronic diseases such as diabetes mellitus and systemic arterial hypertension. The pain symptoms related to endometriosis cause a huge impact on quality of life and can be controlled with the use of these medications [4, 5, 12].

It is natural for patients with endometriosis and pelvic pain to receive medication therapy until there is a desire for reproduction or menopause [4]. Thus, studies like this one, which seek to evaluate the efficacy and tolerability of the medications, are of extreme value.

In the most recent recommendations for endometriosis management, we find a trend towards patient-focused treatment, their desires and symptoms, rather than endometriotic lesions, so medication therapy can be implemented without delay, even in the absence of histological confirmation of the disease [4]. Despite this approach gaining strength, all the patients in the present study obtained a diagnostic confirmation through surgery and a large part of them only started to be followed by the specialized sector after the procedure.

Given the wide range of clinical manifestations and differential diagnoses, the time between the onset of symptoms and the definitive diagnosis with specialized endometriosis group monitoring is usually long [27–29]. This study found an average of 4.8 years between these two events, with a non-parametric distribution, so the median of 2.5 years should be considered. In some scientific articles, the interval described is 6 to 8 years [9, 10, 27]. The two most frequent symptoms were dysmenorrhea and dyspareunia, as reported by other studies [5, 30].

The ovaries are the most affected areas by endometriosis, as shown in this study, which demonstrated ovarian endometriotic foci in 65.64% of the procedures. Definitive surgical treatments, such as hysterectomy and bilateral salpingo-oophorectomy, were exclusion criteria. Among the conservative approaches performed, the resection of endometrioma capsules (48.05%) and unilateral oophorectomies (25.81%) were the most frequent.

The two most prescribed drug classes were combined contraceptives and isolated progestins, in their various doses and administration routes. There is no evidence that demonstrates superiority in pain control by a specific administration route. Considering the prolonged use of these medications, the administration route should facilitate treatment adherence and be in accordance with each patient's preferences [9, 17].

Continuous administration was preferred over cyclic administration for better control of dysmenorrhea. Periodic pauses were indicated only to control irregular bleeding and spotting.

Combined contraceptives and progestins seem to have similar effectiveness in controlling pain symptoms, achieving this result in two-thirds of patients [5, 17, 21].

According to Vercellini et al., combined contraceptives containing the lowest possible dose of ethinylestradiol and second-generation progestin can be considered first-line therapy in peritoneal lesions and endometriomas. Isolated progestins can be prescribed as an alternative to combined contraceptives when there are side effects, deep endometriosis, and in case of a contraindication to estrogen use, such as a high risk of thromboembolism [7, 17, 31]. In fact, the group that received isolated progestins as a first-line therapeutic option was associated with a more advanced age and personal history of chronic diseases.

The most frequent adverse event among patients who discontinued the proposed treatment were breakthrough bleedings, present in 77.08% of cases. Breakthrough bleedings were found in both groups and with a direct and independent association with medication interruption. This finding suggests that proper management of breakthrough bleedings may impact adherence and, consequently, therapeutic success.

Pain persistence is expected in some patients using first-line therapies such as combined contraceptives and progestins. Some authors report a lack of response to these medications in up to 30% of patients, a therapeutic failure attributed to the progesterone resistance present in the disease's pathophysiology [17, 32]. In this study, pain persistence was significantly correlated with treatment interruption only in patients using EP.

EP treatments were significantly correlated with discontinuation, and this risk was higher in the first nine months of treatment. They are widely used to control symptoms of endometriosis, but various authors question their benefits. Some, for example, cite the lack of response in pain outside the menstrual period and dyspareunia, as well as the suspicion that EPs may induce the progression of endometriotic lesions [32–34].

The decrease in the rate of EP interruption over the months may represent patients' adaptation to the side effects of these drugs, so if there is resilience and good guidance on side effects at the beginning of treatment, the chances of good tolerability are higher.

It is necessary to highlight that all patients underwent some surgical treatment, which allowed the histological diagnosis to be used as an inclusion criterion for the study. Thus, the benefits acquired by surgery should be considered.

The choice of medical therapy for endometriosis is not simple. Several factors must be evaluated, such as main symptoms, reproductive desire, types of lesions found, side effects, comorbidities, and personal preferences of the patient. The importance of the patient's reception by the medical team with proper management of complications must also be emphasized.

## Conclusion

The treatment with combined contraceptives has been associated with a higher risk of discontinuation than treatment with isolated progestins. This risk was significantly higher in the first 9 months of treatment. Among all the described complaints, breakthrough bleeding, weight gain, and persistence of pelvic pain had a direct, significant and independent association with medication discontinuation.

## Data Availability

The authors confirm that the data supporting the findings of this study are available within the article. Raw data that support the findings of this study are available from the corresponding author, upon request.
